# Impact of physical exercise programs in breast cancer survivors on health-related quality of life, physical fitness, and body composition: Evidence from systematic reviews and meta-analyses

**DOI:** 10.3389/fonc.2022.955505

**Published:** 2022-12-09

**Authors:** Ana Joaquim, Inês Leão, Pedro Antunes, Andreia Capela, Sofia Viamonte, Alberto J. Alves, Luísa A. Helguero, Ana Macedo

**Affiliations:** ^1^ Department of Medical Oncology, Centro Hospitalar de Vila Nova de Gaia/Espinho, Vila Nova de Gaia, Portugal; ^2^ ONCOMOVE, AICSO – Associação de Investigação de Cuidados de Suporte em Oncologia, Vila Nova de Gaia, Portugal; ^3^ Institute of Biomedicine (IBIMED), University of Aveiro, Aveiro, Portugal; ^4^ Research Center in Sport Sciences, Health, and Human Development (CIDESD), University of Beira Interior, Covilhã, Portugal; ^5^ Centro de Reabilitação do Norte, Centro Hospitalar de Vila Nova de Gaia/Espinho, Vila Nova de Gaia, Portugal; ^6^ Department of Medical Sciences, University of Aveiro, Aveiro, Portugal; ^7^ Research Center in Sports Sciences Health Sciences and Human Development (CIDESD), University of Maia, Maia, Portugal; ^8^ Faculdade de Medicina e Ciências Biomédicas, Universidade do Algarve, Faro, Portugal; ^9^ Medical Education Department, Evidenze Group, Lisboa, Portugal

**Keywords:** breast cancer, physical exercise, systematic review, meta-analysis, quality of life

## Abstract

**Background:**

Breast cancer is the most common cancer worldwide, and despite remarkable progress in its treatment, the survivors’ quality of life is hampered by treatment-related side effects that impair psychosocial and physiological outcomes. Several studies have established the benefits of physical exercise in breast cancer survivors in recent years. Physical exercise reduces the impact of treatment-related adverse events to promote a better quality of life and functional outcomes.

**Aim:**

This study aims to provide an overview of systematic reviews and meta-analyses on the effect of physical exercise on the health-related quality of life, cardiorespiratory fitness, muscle strength, and body composition of breast cancer survivors.

**Methods:**

PubMed and Cochrane databases were searched for systematic reviews and meta-analyses from January 2010 to October 2022. The main focus was ascertaining the effectiveness of physical exercise in breast cancer survivors undergoing curative treatment (surgery and/or radiotherapy and/or chemotherapy). Two reviewers independently screened the literature, extracted the data, and assessed the risk of bias in the included studies.

**Results:**

A total of 101 studies were identified, and 12 were yielded for final analysis. The eligible studies included nine systematic reviews/meta-analyses, one meta-analysis/meta-regression, and two systematic reviews. The number of randomised clinical trials included in each review varied from 11 to 63, and the number of participants was from 214 to 5761. A positive and significant effect of different physical exercise interventions on health-related quality of life was reported in 83.3% (10 studies) of the eligible studies. Physical exercise also improved cardiorespiratory fitness (3 studies; 25%) and showed to be effective in reducing body weight (3 studies; 25%) and waist circumference (4 studies; 33.3%).

**Conclusions:**

Our results suggest that physical exercise is an effective strategy that positively affects breast cancer survivors’ quality of life, cardiorespiratory fitness, and body composition. Healthcare professionals should foster the adoption of physical exercise interventions to achieve better health outcomes following breast cancer treatments.

**Systematic review registration:**

https://inplasy.com/inplasy-2022-11-0053/, identifier INPLASY2022110053.

## Introduction

Breast cancer (BC) is women’s most prevalent diagnosed malignancy, representing the most common cause of cancer-related death worldwide (1). Indeed, BC was responsible for around 16% of worldwide cancer deaths in women in 2020, and by 2040 the incidence is expected to increase by more than 46% (corresponding to one million deaths per year) (2–4). Although a significant increase in the incidence of BC has been detected in recent decades, the mortality rate follows an inverse trend, mainly due to the adoption of preventive measures, early screening, and advances in anticancer therapies (4).

Despite the remarkable progress in BC clinical management, the journey of BC patients after curative treatment can be hampered by chronic issues such as reduced health-related quality of life (HRQoL), reduced physical fitness and body composition alterations (5–7).

The HRQoL of the survivors is affected by treatment-related side effects that impair psychosocial and physiological outcomes (8–10). Each therapeutic approach has specific adverse effects that may compromise the HRQoL, namely surgery (radical or partial mastectomy, with or without reconstruction, with or without removal of lymph nodes), radiotherapy and several modalities of systemic treatment, such as chemotherapy, hormone therapies and other target therapies (11). Evidence emphasises the benefits of physical exercise (PE) on the HRQoL of BC survivors. Indeed, prescribing PE twice or thrice a week improves patients’ HRQoL and health status (12, 13). However, the level of evidence is still low to moderate on this topic (6).

Two components of physical fitness are cardiorespiratory fitness and muscle strength. Cardiorespiratory fitness, measured as maximal oxygen consumption (VO_2_ max), is a good measure of the impairment caused by cardiovascular disease. It has also been shown to be lower in BC survivors compared with healthy women, and this reduction is most pronounced after post-adjuvant treatment, which is related to multiple factors (14). BC survivors are typically characterised as possessing risk factors for cardiovascular diseases and an inappropriate lifestyle, including sedentarism (15–17). In addition, one of the significant challenges in clinical practice is cardiotoxicity (17, 18), which is mainly associated with exposure to BC traditional cytotoxic therapies, such as anthracyclines and anti-human epidermal growth factor receptor 2 (HER2) therapies (19, 20). PE is an essential component of cardiac rehabilitation for adults with cardiovascular diseases. Moreover, observational studies also indicate that PE reduces the risk of subsequent chronic diseases, such as cardiovascular ones (21). Still, there is insufficient evidence of knowledge on the PE effect on cardiac outcomes of BC survivors.

BC survivors suffer from fatigue in 90% of cases, not only during chemotherapy (22) but over a period that may endure for several years (23). Importantly, cancer-related fatigue often elicits a vicious circle of fatigue-induced reductions in PE, causing a significant reduction in muscle mass and muscle strength (including upper and lower limbs) (24). Isometric handgrip maximal strength can be used as an indicator of overall muscle strength, and low values are related to increased all-cause and cancer mortality, including in BC (25, 26). Recent meta-analyses confirmed the effectiveness of exercise in reducing cancer-related fatigue during and after treatment and improving lower body strength, upper body strength, and lean mass during chemotherapy and radiotherapy in patients with cancer (27, 28). A randomised controlled trial (RCT) also showed that resistance training improves upper and lower body maximal muscle strength in older postmenopausal BC survivors, although improvements were not extended to handgrip strength (29).

The journey of most BC survivors is characterised by increases in body weight and waist circumference concerning several factors, such as sedentarism (30–33), emotional stress, mainly depression and anxiety (34, 35), and premature menopause (36). In most BC survivors whose weight is increased after BC diagnosis, the risk of recurrence and death from BC is significantly higher than in normal-weight women (37, 38). Therefore, regular PE can significantly assist in controlling body weight and has already been shown to reduce the risk of BC (39).

Although there are various systematic reviews and meta-analyses on the effect of PE in BC patients on several different outcomes, none focused explicitly on HRQoL, physical fitness and body composition. This study aims to provide an overview of systematic reviews and meta-analyses on the effect of PE in BC patients after curative treatment on HRQoL, physical fitness (cardiorespiratory fitness and isometric handgrip maximal strength) and body composition (body weight and waist circumference).

## Materials and methods

The present overview of systematic reviews and meta-analyses was conducted following the Preferred Reporting Items for Systematic Reviews and Meta-analyses (PRISMA) guidelines (40, 41) and was registered in INPLASY (identifier INPLASY2022110053 and DOI number 10.37766/inplasy2022.11.0053).

### Search strategy

A systematic literature search was conducted in PubMed and Cochrane databases from January 2010 to October 2022. The systematic search used the following keywords: (breast cancer), (effectiveness OR efficacy OR effective*), (Exercise OR Physical Activity OR Strength Training OR Strength Exercise OR Resistance Training OR Resistance Exercise OR Weight Training OR Weight Exercise OR Aerobic Training OR Aerobic Exercise OR Endurance Training OR Endurance Exercise OR Combined Training OR Combined Exercise), “Meta-Analysis”, and “Systematic Review”. The search strategy has been included as **Supplementary Information**.

### Eligibility criteria and study selection

For this systematic analysis, were included only systematic reviews and meta-analyses described in full-length articles in English with clinical observations of humans, with a clearly defined clinical question, details of inclusion and exclusion criteria, details of searched databases and relevant search strategies, and a summary of results, per group, for at least one of the desired outcomes. **Table 1** reports the inclusion criteria of the study Population, Intervention, Comparator, Outcomes, and Settings (PICOS). Eligibility screening was performed through two separate steps: a) titles and abstracts screening and b) full texts screening, and by three independent persons. Each study title and/or full text was screened by two independent reviewers, and discrepancies were excluded. All the retrieved articles were used independently of their outcome (positive, negative, or neutral impact of the physical exercise programs on health-related quality of life, physical fitness, and body composition of breast cancer survivors).

**Table 1:** Study inclusion criteria, defined by PICOS.

PICOS Component	Description
**Population**	Adults (age >18y, male or female) breast cancer survivors who have concluded curative treatment (surgery and/or radiotherapy and/or chemotherapy) at least 1 month before the intervention. They could be on hormone therapy (any type) and/or anti-HER2 drugs
**Intervention**	Individual PE interventions or in groups, supervised or unsupervised/home-based (including PE that could be initially taught by an exercise professional, or involve periodical/ongoing supervision), and examining different modes of exercise (aerobic exercise, resistance exercise, and combined exercise)
**Comparator**	No intervention or standard care
**Outcomes**	HRQoL (measured by validated questionnaires); cardiorespiratory fitness (measured by exercise stress test); isometric handgrip maximal strength (assessed by dynamometry); body composition (body weight, BMI and/or waist circumference)
**Setting**	Systematic reviews and meta-analyses

BMI, body mass index; HRQoL, health-related quality of life; PE, physical exercise.

### Data extraction

Two independent reviewers extracted the relevant data from the included studies using a preformatted data extraction sheet. The extracted data included: a) baseline characteristics of the population; b) baseline characteristics of the study as design, sample size, procedure evaluation and used comparators; c) assessed outcomes; d) meta-analyses results; and e) conclusions of the study.

### Risk of bias assessment

Included systematic reviews were assessed by two researchers for risk of bias using the Assessment of Multiple Systematic Reviews v2 (AMSTAR-2) (42). Disagreements in the scoring were solved through discussion and consensus. The following seven AMSTAR-2 domains were considered critical: item 2: review methods established before conducting the review; item 4: comprehensive literature search; item 6: data extraction in duplicate; item 9: risk of bias satisfactorily assessed; item 11: appropriate methods for statistically combining results; item 13: risk of bias considered when interpreting/discussing review results; and item 15: quantitative synthesis – adequate investigation of publication bias (slight study bias) and discussion. An overall rating of confidence in the results of each review, of high, moderate, low, or critically low, was given. This depended on the flaws in the above critical domains or other weaknesses within the systematic review. The quality of the preliminary randomised clinical trials (RCTs), as judged by the authors of the included systematic reviews, was considered, particularly random sequence generation, allocation concealment, and attrition bias.

## Results

**Figure 1:**
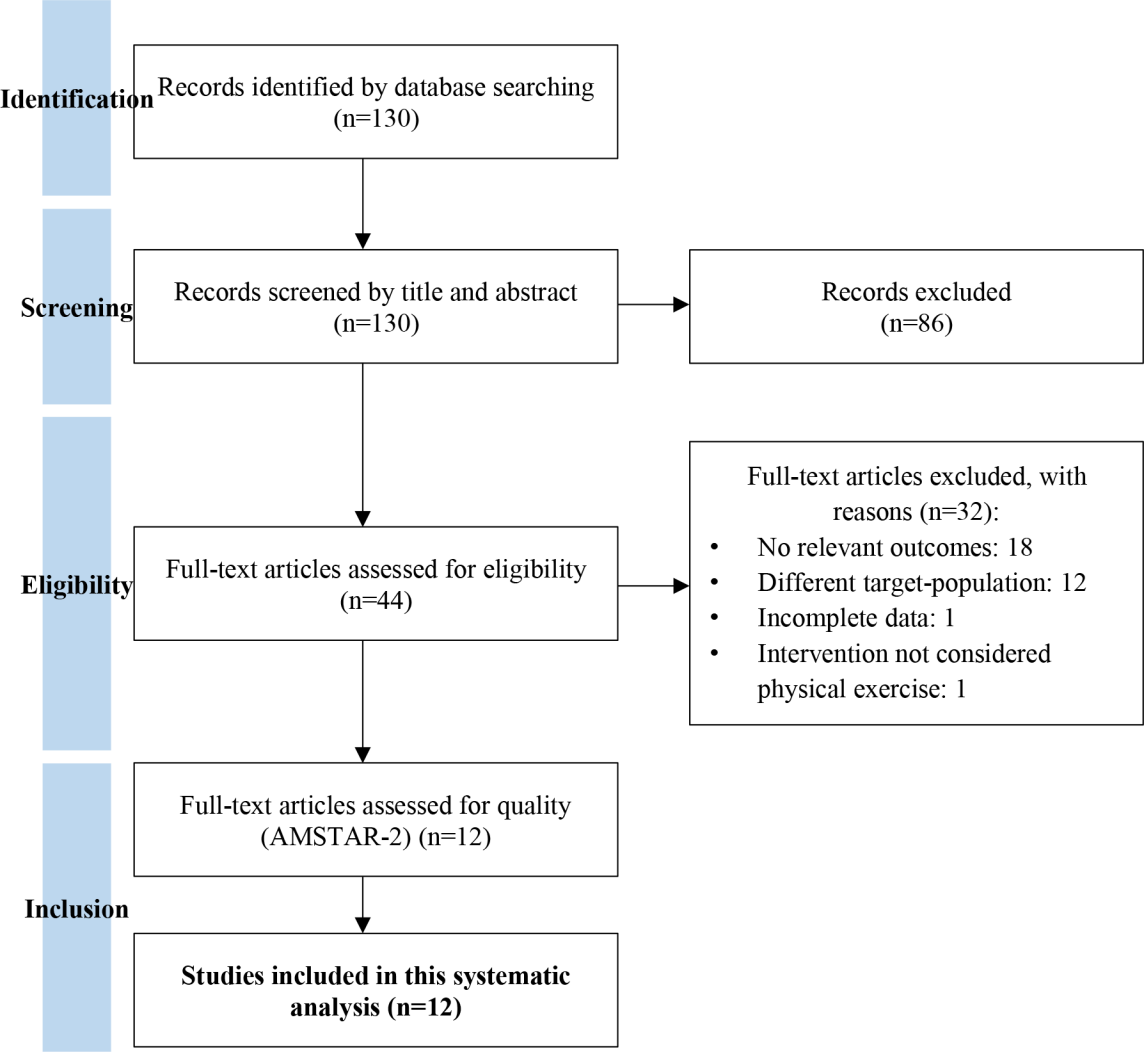
PRISMA flow chart of study selection (41).

The literature search on PubMed and Cochrane databases yielded 130 studies meeting the search criterion, with 12 studies included in the final version (**Figure 1**, **Table 2**). During the initial screening, 86 records were excluded based on title and abstract. During the second phase of the selection process of the remaining 44 reports, 32 records were excluded because they were not eligible for this study due to not having relevant outcomes (18 reports), having a different target population (12 reports), having incomplete data (1 reports) or for having an intervention not considered PE (1 report). The remaining 12 articles were subjected to quality assessment by AMSTAR-2 (42), considering seven critical domains (**Table 3**) and included in this systematic analysis. The methodological quality and the overall quality of the studies were low (1 study), moderate (6 studies) and high (5 studies).

**Table 2:** Included systematic reviews and meta-analyses methodology and results.

Study	Objectives & Population	Procedure under evaluation (N)/Comparator(s)/Type and number of studies	Primary outcomes	Meta-Analysis: results, comparison values, and significance	Conclusions
Boing, 2020 (43)	Investigate PE effect on physical outcomes in BC women receiving any modality of hormone therapy.	Effect of PE on physical outcomes (N=368)/ PE *vs* usual care; Unsupervised PE *vs* supervised PE/ 3 RCTs; 2 single-arm pilot studies.	Cardiorespiratory fitness (VO_2_max), muscle strength, pain, body fat percentage, bone mineral density.	Cardiorespiratory fitness: SMD = 0.37; p=0.005. Grip strength: SMD = 0.298; p=0.091.	Three of the five trials demonstrated significant effects separately in improving VO_2_max. This trend was reflected in the meta-analysis.
Abdin, 2019 (44)	Evaluate PE and specifically consider the effects of different types of exercise and intervention (group *vs* individual) in adult patients with BC (invasive and *in situ* carcinoma).	Intervention aiming to increase PE (N=2208)/ Different types of PE interventions/ 17 RCTs.	Self-reported levels of PE, adherence, cardiorespiratory fitness, QoL, BMI, weight, and fatigue.	Meta-analysis was not performed (due to population heterogeneity, intervention components, outcome measurements, and duration of interventions).	Individual and group interventions have positive outcomes, but some indicators highlight more benefits in group interventions. It was impossible to conclude whether there are differences in outcomes depending on the type of PE. It remains apparent that the lack of clarity of reporting and theory in intervention design is a problem.
Hong, 2019 (45)	Examine PE effect on HRQoL, social function, and physical function; explore the most effective characteristics of PE (type, frequency, duration, time, and total exercise time); and determine optimal PE time for HRQoL improvement in adults diagnosed with BC.	PE intervention (aerobic, resistance, combined, yoga, and Qigong) (N= 1892 in the systematic review; N=1205 [exercise 602 and control 603])/ Not submitted to PE intervention/ 26 RCTs in the systematic review and 18 in the meta-analysis.	QoL (general, global health, and overall QoL), SF, and PF.	Change in HRQoL: extremely (p = 0.0004) influenced by exercise intervention, with heterogeneity: Tau2 = 0.10; Chi2 = 43.68; df = 17; and I2 = 61%. “Time of session”: significantly (p = 0.041) correlated with an improved QoL. SF outcome: extremely favoured exercise, citing the SMD = 0.20, I2 = 16%, and 95% CI: 0.08 to 0.32. PF outcome: improved by exercise interventions (p < 0.00001); pooled SMD of the enhanced PF was 0.32 (0.20 to 0.44), at a 95% CI, and where the I2 was 32% after the interventions.	PE interventions (of any type) improve HRQoL and social and physical function in women BC survivors. However, HRQoL improvement was associated with session duration (>45 min).
Soares Falcetta, 2018 (46)	Disclose PE effect (with or without dietary interventions) on body composition, HRQoL, and survival in women after early-stage (I–III) BC treatment.	Studies that performed the intervention after the end of adjuvant treatment (excluding hormone therapy) were included; studies that applied the intervention after 5 years from the diagnosis were excluded. From a total of 60 studies included, only 19 RCTs with a structured or individualised PE program (N=1613; PE 835 and control 778) were assessed.	Overall survival and disease-free survival (5 years after treatment or until the maximum follow-up study). Secondary endpoints: weight loss (kg), BMI (kg/m^2^), waist-hip ratio, percentage of body fat (%), and HRQoL. AEs, such as PE-induced lesions, were also considered.	Weight reduction: mean diff -0.27 (-1.16;0.63); n=835 (experimental group) and n=787 (control group); BMI reduction: -0.36 (-0.83;0.11); n=797 (experimental group) and n=752 (control group); HRQoL (general) for different scales: SMD=0.76 (0.19-1.34); n= 421 (experimental group) and n=388 (control group).	Heterogeneous types of intervention showed significant effects on anthropometric measures and HRQoL. Only one study had mortality as an outcome, showing PE as a protective intervention. Despite these findings, publication bias and poor methodological quality were presented. PE should be advised for BC survivors since it has no AEs and can improve anthropometric measures and QoL.
Singh, 2018 (47)	Evaluate PE safety, feasibility, and effect among women with stage II+ BC.	Randomised, controlled PE trials were included, involving at least 50% of women diagnosed with stage II+ BC. From the 61 trials included in this systematic review, 60 RCTs evaluated PE safety and the risk of AEs.	The risk of bias was assessed, and AEs severity was classified using the Common Terminology Criteria. Feasibility was evaluated by computing median (range) recruitment, withdrawal, and adherence rates. Meta-analyses were performed to evaluate PE safety and effects on health outcomes only. The influence of intervention characteristics (mode, supervision, duration, and timing) on PE outcomes were also explored.	Significant effects of PE on HRQoL, fitness, fatigue, strength, anxiety, depression, BMI, and waist circumference compared with usual care (stand mean diff range: 0.17-0.77, p<0.05). There were no differences in AEs between PE and usual care (risk difference: <0.01 ([95% CI: -0.01, 0.01], p=0.38). The median recruitment rate was 56% (1%-96%), the withdrawal rate was 10% (0%-41%), and the adherence rate was 82% (44%-99%). Safety and feasibility outcomes were similar, irrespective of PE mode, supervision, duration, or timing.	The findings support PE safety, feasibility, and effects for those with stage II+ BC, suggesting that national and international exercise guidelines appear generalisable to women with local, regional, and distant BC.
Lahart, 2018 (6)	Assess the effects of PE interventions after adjuvant therapy for women with BC.	Randomised and quasi-randomised trials comparing PE interventions *vs control* (e.g., usual or standard care, no PE, no exercise, attention control, placebo) after adjuvant therapy (i.e., after completion of chemotherapy and/or radiation therapy, but not hormone therapy) in women with BC. The study included 63 trials that randomised 5761 women to a physical activity intervention (n = 3239) or a control (n = 2524).	Outcomes of HRQoL, PE, and cardiorespiratory fitness. The overall effect size with 95% CIs was calculated for each outcome; GRADE was used to assess the quality of evidence for the most critical outcomes. GRADE working group grades of evidence: High quality: Further research is unlikely to change the confidence in the effect estimate. Moderate quality: Further research is likely to impact the confidence in the estimate of effect and may change the estimate. Low quality: Further research is very likely to impact the confidence in the estimate of effect and is likely to change the estimate. Very low quality: There is high uncertainty about the estimate.	Changes from baseline to the end of intervention after a median follow-up of 12 weeks: 1 – HRQoL: 14 studies were assessed, comprising 1459 participants. The illustrative comparative risk (95% CI) was 0.78 standard deviations higher (0.39 to 1.17 higher) in the physical activity group and -2.40 to 1.25 standard deviation units in the control group. Quality of evidence (GRADE): low. 2 – Emotional function/mental health: 15 studies were assessed, comprising 1579 participants. The illustrative comparative risk (95% CI) was 0.31 standard deviations higher (0.09 to 0.53 higher) in the physical activity group and -0.39 to 3.47 standard deviation units in the control group. Quality of evidence (GRADE): low. 3 – Perceived physical function: 13 studies were assessed, comprising 1433 participants. The illustrative comparative risk (95% CI) was 0.60 standard deviations higher (0.23 to 0.97 higher) in the physical activity group and -1.34 to 1.66 standard deviation units in the control group. Quality of evidence (GRADE): moderate. 4 – Anxiety change: 4 studies were assessed, comprising 235 participants. The illustrative comparative risk (95% CI) was 0.37 standard deviations lower (0.63 to 0.12 lower) in the physical activity group and -1.44 to 0.73 standard deviation units in the control group. Quality of evidence (GRADE): low. 5 – Depression change: 7 studies were assessed, comprising 816 participants. The illustrative comparative risk (95% CI) was 0.34 standard deviations lower (0.63 to 0.05 lower) in the physical activity group and -1.51 to 1.83 standard deviation units in the control group. Quality of evidence (GRADE): low. 6 – Fatigue change: 13 studies were assessed, comprising 1289 participants. The illustrative comparative risk (95% CI) was 0.30 standard deviations lower (0.61 to 0 lower) in the physical activity group and -1.81 to 1.83 standard deviation units in the control group. Quality of evidence (GRADE): low. 7 – Cardiorespiratory change: 9 studies were assessed, comprising 863 participants. The illustrative comparative risk (95% CI) was 0.83 standard deviations higher (0.40 to 1.27 higher) in the physical activity group and -1.45 to 2.38 standard deviation units in the control group. Quality of evidence (GRADE): very low.	There were no conclusions regarding BC-related and all-cause mortality or BC recurrence. However, PE interventions may have small-to-moderate beneficial effects on HRQoL, emotional or perceived physical and social function, anxiety, cardiorespiratory fitness, and self-reported and objectively measured physical activity. The positive results reported in the current review must be interpreted cautiously owing to the very low-to-moderate quality of evidence, heterogeneity of interventions and outcome measures, imprecision of some estimates, and risk of bias in many trials. Future studies with a low risk of bias are required to determine the optimal combination of physical activity modes, frequencies, intensities, and durations needed to improve specific outcomes among women who have undergone adjuvant therapy.
Zhang, 2019 (48)	Assess the PE effect on the HRQoL among people with BC.	Effect of PE on HRQoL compared with that of usual care for people with BC. N = 36 RCT (3914 participants)	PE was categorised into three modes: aerobic, resistance, and a combination of aerobic and resistance. The outcome measure was the QoL.	Meta-analysis was not performed. This Systematic review revealed that all three PE intervention modes significantly affected the QoL between groups.	PE is a safe and effective way to improve HRQoL in BC patients. Combined training was associated with a significant improvement in HRQoL. In future research, more high-quality, multicenter trials evaluating the effect of exercise in BC patients are needed.
Gebruers, 2019 (49)	Characterise PE programs and their effects on (1) physical performance outcomes, (2) experienced fatigue, and (3) HRQoL in patients during the initial treatment for BC.	N = 28 RCT (2525 participants)	The primary outcome was the PE effect on physical performance, HRQoL, and perceived fatigue.	Meta-analysis was not performed.	Most training interventions provided an improvement in physical performance and a decrease in perceived fatigue. HRQoL was the outcome variable least susceptible to improvement.
Kannan, 2022 (50)	Investigate PE effect on QoL and upper quadrant pain in women with PMPS (post-mastectomy pain syndrome)	PE intervention (aerobic exercise, resistance training, aqua fitness) No PE intervention N=10 RCT (4 RCT with 451 patients to evaluate PE effect on QoL; 6 RCT with 406 patients to evaluate PE effect on upper quadrant pain)	QoL, upper quadrant pain	Effect of PE on QoL: a statistically significant effect of the intervention on general [SMD 0.87 (95%CI: 0.36-1.37); p = 0.001], physical [SMD 0.34 (95%CI: 0.01-0.66); p = 0.044] and mental health components [SMD 0.27 (95%CI: 0.03-0.51); p = 0.027], when compared to the control condition. Effect of PE on upper quadrant pain: more significant reduction in pain severity in the intervention group than the control group [SMD -1.00 (95%CI: -1.48 to -0.52); p < 0.001)	Meta-analysis revealed statistically significant effects of exercise compared to control in improving overall QoL and pain. Exercise is a low-cost and safe intervention and could, therefore, be considered an essential component of QoL and pain management among women with PMPS
Salam, 2022 (51)	Evaluate the effects of post-diagnosis PE on depression, physical functioning, and mortality in breast cancer survivors	PE intervention (home-based/unsupervised; supervised aerobic resistance, strengthening and core exercises, yoga, gymnastics) No physical activity (i.e., a regular care group or non-physical intervention) N=26 RCT (13 studies for the effect of PE on depression, 8 for the effect of PE on physical functioning/QoL, 7 for the effect of PE on mortality; some studies were investigating both depression, physical functioning, and mortality, therefore, entered twice for the statistical analysis)	Depression (measurements with CES-D, HADS, BDI, POMS and Greene Climacteric Scale), physical functioning/QoL (SF-36 and EORTC QLQ-C30 subscales), mortality	Effect of PE on depression (N= 689 participants in the PE group vs 480 participants in the control group): differences in the depression scores were statistically significant compared with controls (SMD -0.24, 95% CI -0.43 to -0.05, P = 0.012), with moderate statistical heterogeneity identified (P = 0.011, I^2^ = 54%) Effect of PE on physical functioning/QoL (N= 689 participants in the PE group vs 480 participants in the control group): statistically significant differences between the PE and control groups (SMD 0.37, 95% CI 0.03-0.72, P = 0.032), with moderate statistical heterogeneity (P = 0.01, I^2^ = 62%) Effect of PE on mortality (N=15,853 participants): the overall effect of physical activity was statistically significant (HR 0.63, 95% CI 0.55-0.71, p < 0.00001), with no evidence of statistical heterogeneity (p = 0.27, I^2^ = 15%).	There is sufficient evidence to support the effectiveness of PE and physical activity in addressing cancer-related health outcomes, including fatigue, quality of life, physical function, anxiety, and depressive symptoms
Wang, 2022 (52)	Evaluate the benefits of aquatic physical therapy as a rehabilitation strategy for women with BC	Aquatic exercise (8 weeks) Usual care and all forms of intervention except aquatic exercise N=2 RCT (52 patients on PE-group vs 51 patients in the control group)	Fatigue, waist circumference	Effect of aquatic PE on fatigue: statistically significant differences among groups (MD = -2.14, 95% CI: -2.82, -1.45, p<0.01), with 0% of heterogeneity. Effect of aquatic PE on waist circumference: no statistically significant differences between groups (MD = -3.49, 95% CI: -11.56, 4.58, p = 0.4)	Aquatic physical therapy significantly relieved fatigue. However, compared with usual care, aquatic physical therapy did not improve physical index (waist circumference), which might be due to the short intervention time, which is not enough to produce a significant statistical difference
Ye, 2022 (53)	Investigate the effects of Baduanjin exercise on the QoL and psychological status of postoperative patients with BC	Baduanjin exercise No PE (i.e., a regular care group or non-physical intervention) N=7 RCT (450 participants)	QoL (measurements with FACT-B and SF-36 scores), anxiety (measurements with SAS and SDS scales)	Effect of Baduanjin on QoL (FACT-B): Exercise-group with higher values of QoL than the control group (WMD with 95% CI = 5.70 (3.11, 8.29), P < 0.0001) Effect of Baduanjin on QoL (SF-36): PE improved QOL in the dimensions of role-physical (WMD with 95% CI = 11.49 [8.86, 14.13], P < 0.00001, I^2^ = 0%) and vitality (WMD with 95% CI = 8.58 [5.60, 11.56], P < 0.00001, I2 = 0%), but no statistical difference was found for physical functioning, bodily pain, social functioning, general health, and mental health (physical functioning: WMD with 95% CI = 0.97 (−1.57, 3.50), P = 0.45, I^2^ = 0%; bodily pain: WMD with 95% CI = 0.81 (−1.97, 3.58), P = 0.57, I^2^ = 0%; social functioning: WMD with 95% CI = −0.50 (−16.91, 15.90), P = 0.95, I^2^ = 62%; general health: WMD with 95% CI = 2.97 (−0.05, 5.99), P = 0.05, I^2^ = 0%; role-mental: WMD with 95% CI = 3.03 (−3.18, 9.24), P = 0.34, I^2^ = 5%; mental health: WMD with 95% CI = 7.47 (−1.01, 15.94), P = 0.08, I^2^ = 75%). Effect of Baduanjin on anxiety: depression scores for the exercise group were lower than those of the control group (WMD with 95% CI = -4.45(-5.62, -3.28), P < 0.00001).	Results showed that Baduanjin interventions improved the QOL of postoperative patients with BC compared to those without Baduanjin. Subgroup analysis found that Baduanjin exercise improved physical function and vitality in postoperative patients with BC. In terms of anxiety and depression relief, Baduanjin exercise also had a significant effect.

BC, Breast cancer; PE, Physical exercise; AEs, Adverse events; BMI, Body Mass Index; SMD, standardized mean difference; HRQoL, Health-Related Quality of life; RCT, randomized clinical trials; SF, social function; PF, physical function; df, degrees of freedom; HRQoL, Health-related quality of life.

**Table 3:** Quality assessment (AMSTAR-2) of the included systematic reviews and meta-analyses.

AMSTAR 2 criteria*	Boing, 2020	Abdin, 2019	Hong,2019	Falcetta,2018	Singh,2018	Lahart,2018	Zhang,2019	Gebruers,2019	Kannan, 2022	Salam, 2022	Ye,2022	Wang, 2022
**Item 2**	Y	Y	PY	Y	PY	Y	PY	Y	PY	Y	Y	Y
**Item 4**	Y	Y	Y	Y	Y	Y	Y	Y	Y	Y	Y	Y
**Item 6**	Y	Y	?	?	PY	Y	PY	Y	Y	Y	Y	Y
**Item 9**	Y	Y	Y	Y	Y	Y	Y	Y	Y	Y	Y	Y
**Item 11**	Y	NMC	?	Y	Y	Y	NMC	NMC	Y	Y	Y	Y
**Item 13**	Y	Y	Y	Y	PY	Y	Y	Y	Y	PY	Y	Y
**Item 15**	PY	Y	Y	Y	Y	Y	PY	Y	Y	PY	Y	Y

N, no; NMC, no meta-analysis conducted; PY, partial yes; Y, yes.

*AMSTAR-2 Criteria (42):

Item 2: Did the review report explicitly state that the review methods were established before conducting the review, and did the report justify any significant deviations from the protocol?

Item 4: Did the review authors use a comprehensive literature search strategy?

Item 6: Did the review authors perform data extraction in duplicate?

Item 9: Did the review authors use a satisfactory technique for assessing the risk of bias (RoB) in individual studies included in the review?

Item 11: If meta-analysis was justified, did the review authors use appropriate methods for statistical.

Item 13: Did the authors consider RoB in individual studies when interpreting/discussing the review results?

Item 15: If they performed quantitative synthesis, did the review authors carry out an adequate investigation of publication bias (slight study bias) and discuss its likely impact on the results of the review?

### Description of included studies

The studies selected for this review consisted of 9 systematic reviews/meta-analyses, 1 meta-analysis/meta-regression, and 2 systematic reviews. Of the 12 included studies, 3 were conducted across Europe (2 from the United Kingdom and 1 from Belgium), 2 in South America (Brazil), 4 in Asia (5 from China and 1 from Saudi Arabia), and 1 in Australia. Overall, the number of databases used in each review ranged from 4 to 10, the number of studies included varied from 5 to 63, and the number of participants was from 214 to 5761.

#### Outcomes analysis

The detailed summary and primary outcomes of the eligible studies are presented in **Table 2**. Overall, the studies herein analysed depicted a positive and significant effect of different PE interventions on the health outcomes of BC survivors, namely on HRQoL (10 studies; 83.3%), improved cardiorespiratory fitness (3 studies; 25%), reducing bodyweight (3 studies; 25%) and waist circumference (4 studies; 33.3%).

##### Health-related quality of life

A meta-analysis published by Hong et al. evaluated 18 trials to assess the effect of PE intervention on the HRQoL of BC survivors and showed that the HRQoL was significantly improved by PE intervention (45). The authors found no relationship between HRQoL and PE characteristics (type, frequency, and total time) except for the exercise session duration. Specifically, the trials with “time of session” data were categorised into 3 subgroups, namely, shorter time (≤ 45 min, 7 trials), medium time (> 45 to ≤ 60 min, 7 trials), and longer time (> 60 to 90 min, 4 trials), and subgroup analysis was performed. Results revealed that the medium and longer-time sessions enhanced the HRQoL of BC patients, the latter further positively associated with increased QoL, as the patients engaged in longer-time sessions achieved the most significant improvement (>60 to 90 min, p = 0.005). In another study that also evaluated HRQoL, Soares-Falcetta et al. analysed 23 studies and reported a positive effect of PE on the improvement of BC patient HRQoL (p < 0.01) (46). The authors also revealed that heterogeneous types of physical intervention benefit the HRQoL in women after early-stage (I-III) BC treatment. Importantly, it was impossible to identify the best one due to the heterogeneity of the PE interventions among the analysed studies, varying from exercise counselling to structured and supervised exercise programs. Hence, the authors compared PE as a single group (46).

The meta-analysis by Singh et al. analysed 40 studies to compare PE (aerobic, resistance, or other - not specified as aerobic or resistance) with usual care and found a moderate pro-exercise effect on HRQoL (p<0.01) (47). Specifically, supervised PE had a more significant beneficial effect on the HRQoL of the participants (p < 0.01), compared to unsupervised interventions, and when the PE involved more than one exercise mode, compared with only one mode.

HRQoL was assessed in 22 studies in Lahart et al. meta-analysis, in which PE induced a small-to-moderate significant improvement in HRQoL compared to the control group (6). The results also indicated that this improvement did not persist for three months or longer after the intervention, which had an overall duration ranging from 4 to 24 months, with most lasting 8 or 12 weeks (37 of 63 RCTs). In a recent systematic review by Gebruers et al., 28 RTCs, comprising 2,525 participants, were analysed (49). Evidence showed that PE intervention improved HRQoL and decreased fatigue in BC survivors (49). In the systematic revision by Zhang et al., the effect of different types of PE on the HRQoL was investigated, particularly aerobic, resistance, and a combination of both (48). The authors concluded that all types of PE were effective in fostering HRQoL of BC survivors, though combined training was associated with a significantly higher improvement. The results suggest that supervised PE enhances attendance rates, motivation, and the overall HRQoL. Likewise, Abdin and colleagues revised 17 RCTs and concluded that both individual and mostly group interventions promote favourable health outcomes and overall HRQoL (44).

The meta-analysis by Kannan et al. also demonstrated a positive effect of different PE interventions on the HRQoL of female survivors of BC with post-mastectomy pain, a common condition among BC survivors (50). For that, the authors evaluated 4 RCTs, comprising 406 women under aerobic exercise (treadmill at an intensity of 60%–80% heart rate maximum), resistance training (exercises for the large muscles of the upper and lower limbs progressing from two sets of 12 repetitions at 50–60% one repetition maximum (RM) to three sets of 10 repetitions at 60–80% 1RM, over 2 years) or aquafitness, in sessions ranging from 30 to 60 min, performed two to five times per week for a duration of 3 months to 2 years. The pooled evidence demonstrated a statistically significant effect (p = 0.001) of these PE interventions on HRQoL.

Similarly, a pool of 8 studies was evaluated by Salam and colleagues (51), combining a total of 241 BC survivors under prescribed PE, ranging from moderate-to-vigorous intensity exercises (e.g. yoga, walking, cycling, tai chi chuan and cycling). When compared to the control group (177 participants under no PE), active participants, in a frequency ranging from once to 6 times/week in 15-to-75 min sessions, displayed significantly better HRQoL (p = 0.032) (51).

Lastly, Baduanjin, a Chinese series of 8 movements combing breathing and body movement, was also demonstrated to improve the psychological status of postoperative patients with BC (53). For that, the authors pooled 7 RCTs, including 450 postoperative BC patients undergoing 30 min-sessions of Baduanjin 2 to 5 times/week, and evaluated the impact on QoL. Both FACT-B (p < 0.0001) and SF-36 (p < 0.00001) scores revealed a significant increase with Baduanjin exercise (53).

##### Cardiorespiratory fitness

The study of Boing et al. analysed the effect of PE on BC survivors receiving hormone therapy, tamoxifen, and aromatase inhibitors (43). This study analysed five RCTs on cardiorespiratory fitness and concluded that three of the five trials separately demonstrated significant effects in improving VO_2_ max, as shown in the meta-analysis (p < 0.01). The Lahart et al. study included 23 RCTs comprising 1265 women and reported that PE significantly increased cardiorespiratory fitness (6). This study also demonstrated that the significant improvement in cardiorespiratory fitness values at post-intervention follow-up was maintained for PE compared with the control in the subgroup analysis only for postmenopausal women, for both aerobic exercise and combined aerobic and resistance exercise interventions (SMD 0.44, 95% CI 0.30 to 0.58, 23 studies, 1265 women, moderate-quality evidence). The analysis performed by Abdin et al. revealed that both individual, but mostly group, interventions had a beneficial effect on health outcomes concerning fatigue and cardiorespiratory fitness (44).

##### Upper body strength

Singh et al. assessed the effect of PE on upper-body strength, and the analysis revealed a moderate effect in favour of exercise, particularly after resistance PE (SMD=0.68 [95%CI: 0.05, 0.85]; p<0.01) rather than aerobic or combined PE (47). Zhang et al. also concluded that PE prescription incorporating more than 150 min of high-intensity training per week significantly improved upper body strength and was associated with a low incidence of adverse events in BC patients (48). Grip strength, defined as the strength that muscles apply against resistance at maximum effort during handgrip, was also assessed in the study by Boing et al. (43). This test is commonly used to verify muscular strength and correlated health in BC survivors, and it showed to be marginally improved in the PE group compared to the control group, though not significantly (43).

##### Body composition

Soares-Falcetta et al. analysed 54 studies addressing the effect of PE, whether by supervision or by structured programs, including aerobic and resistance training, on the reduction of weight and BMI (46). The follow-up ranged from 1 to 101 months, and the duration of the intervention occurred from 4 weeks to 24 months. PE was associated with weight reduction (p=0.02) and lower BMI (p<0.01) (46). In addition, the evidence disclosed by the meta-analysis by Singh et al., in which 8 studies compared PE with usual care, suggests a moderated benefit for waist circumference reduction (SMD=0.22, p=0.03) (47). On the other hand, a sub-analysis of 15 trials showed no significant difference in weight reduction (SMD=0.08, p=0.22) (47), whereas a sub-analysis of 13 trials revealed a slight, though not significant, benefit in BMI (SMD=0.11, p=0.11) (47). Regarding body fat percentage, a meta-analysis by Boing et al., which included 4 RCTs, also reported an overall reduction in body fat after the intervention; however, this reduction was not significant (43).

Finally, aquatic PE was also investigated for a potential effect on the body composition of BC survivors (52), as opposed to conventional land-based exercise. This form of exercise involves a variety of modalities, including aerobic, stretching, resistance, flexibility and stability training, which by using the unique properties of water (buoyancy, resistance, flow, and turbulence), allows people to perform exercises that they cannot do on land (54). A total of 2 RCTs, with 53 participants on aquatic exercise (8 weeks duration of the interventional program) compared to 51 control participants under usual care, were examined for waist circumference. Meta-analysis failed to show significant differences in waist circumference between groups (p = 0.04), which can be due to the low number of participants and the short intervention time, which was insufficient to produce a significant statistical difference. Thus, more studies are required to fully assess aquation PE’s impact on the health outcomes of BC survivors.

## Discussion

In the present review, twelve systematic revisions and meta-analyses were explored to assess the effectiveness of PE in the QoL of BC survivors. The selected studies evaluated many RCTs to disclose how distinct PE methodologies could potentially promote a healthier life, characterised by reduced stress, fatigue, depression, anxiety and an increase in cardiorespiratory fitness. Specifically, the studies examined different modes of exercise (including aerobic exercise, resistance exercise, and combined exercise), types of intervention (individual or group, and supervised or unsupervised/home-based), frequency, and duration of each program training session.

According to the analysis by Zhang and colleagues, the outcome suggests that in almost 90% of the studies, aerobic exercise significantly benefits the QoL in patients with BC compared with the control group (48). Notably, 100% of the studies analysed reported a significant effect of combined training on the QoL in patients with BC compared with the control group (48). The same pattern of benefit in BC patients was observed by Singh et al., as the authors concluded that PE led to favourable effects irrespective of intervention characteristics. Still, the effect’s magnitude depended on the PE mode, degree of supervision, timing (during *vs* following treatment), and duration of the intervention (47). In another study, the same reflection was shared as the authors stated that not only QoL but also social and physical functions in women with BC were improved if longer training sessions occurred (45).

Overall, all the considered reports disclosed a valuable and significant influence of PE on QoL, and although it is unclear whether the type of program or its precise duration influences the results, more prolonged, in-group, and supervised PE sessions seem to have a higher benefit. Practising exercise has been shown to foster the maintenance of a healthy weight and lifestyle, characterised by improved cardiorespiratory fitness and decreased body weight and BMI, fatigue, depression, and anxiety (55). Furthermore, it can also prevent other chronic diseases for which this population is particularly vulnerable (56). Remarkably, PE prescription is more commonly performed to overcome treatment-induced adverse effects and promote general well-being. In numerous trials in which exercise was included, the results indicate that clinical outcomes are advantageous (57), contradicting previous theories whereby cancer patients were advised to rest and avoid physically challenging activities that could promote additional load and fatigue rather than alleviating cancer-related fatigue (58). Aquatic exercise also arises as a PE intervention to be further researched for the prescription to BC survivors due to its therapeutic potential already used on conditions such as fibromyalgia (59) and stroke (56, 60). Considering this new evidence that PE is vital for the long-term management of QoL (61), the interests and preferences of the patients should not be neglected, and any prescription should consider their physical and psychosocial needs. According to some of the studies considered herein, better outcomes were obtained when BC participants were encouraged to train with other survivors or supervised exercise programs (44). Notably, this can potentiate personal interaction with others, fostering the establishment of relationships, decreasing the sense of isolation and social stigma, and improving self-esteem. Overall, PE contributes to lowering withdrawal rates, boosting motivation and adherence, and the long-term commitment to PE, potentiating an improvement in body composition and QoL. Accordingly, despite the mode, frequency, or intervention of the PE program, it has been shown to be quite effective in reducing body weight, fat percentage, and waist circumference, contributing to health outcomes (49). Resistance or intense PE was the most effective in promoting increased upper body strength (48).

Notably, adverse events derived from PE practice were rarely reported, typically mild and representing acute and normal physiological adaptations to exercise. Nonetheless, the authors acknowledge that some studies do not report adverse events. This highlights the need for the standardised recording of adverse events to be incorporated into the design of RCTs, as it is critical to explore the adverse effects of PE on BC patients in future research.

Recent shreds of evidence have also unveiled several biomarkers (e.g., inflammatory molecules, mitochondria modifications) (62, 63) with monitoring potential to improve the clinical rehabilitative management of BC patients. Interestingly, these actionable biomarkers were shown to be modulated by PE (64), opening the possibility of targeted control of the therapeutic effect of PE on BC survivors. Future studies should also envisage deciphering the biological mechanisms by which PE potentiates an improvement of the QoL in BC patients. It would be of great value to disclose whether this enhancement is a consequence of an improved “state of mind” (due to reduction of stress, anxiety, and depression) or is dependent on a biochemical pathway that is disease-related, or both in a complementary way, paving the way for the creation of new approaches to boost the QoL of people who survived cancer.

## Strengths and limitations of the study

In this systematic analysis, we have followed the PRISMA Statement. Although the effect of PE on different outcomes of BC survivors has been increasingly addressed in the literature, this systematic analysis evaluated data focusing specifically on the outcomes of HRQoL, cardiovascular fitness, and increase of upper-body strength and body composition and also includes more recent publications than other published ones. Overall, we showed strong evidence that PE has a potentially positive effect on the HRQoL and health status of BC survivors. These results should instigate the prescription of regular PE programs, after BC diagnosis and survival, by healthcare professionals to enhance survivors’ HRQoL.

Nonetheless, the encouraging results reported herein must be carefully interpreted, as, during the execution of this systematic revision, the authors encountered some difficulties as the multitude of literature on these thematic reports presents highly diverse content, not enabling, for instance, the execution of a meta-analysis. The heterogeneity of interventions and outcome measures, the different comparisons performed, the low to moderate quality of evidence (GRADE scale), the risk of bias in many trials, and the overall lack of long‐term intervention effects do not enable us to draw solid conclusions. Notably, the elaboration of future studies with low-risk bias, improved clarity to describe interventions, and a comparison of data considering the outcomes of PE, is urgently needed. This information will be critical to determine the optimal combination of PE modes, frequencies, intensities, and durations needed to improve specific outcomes among patients who survived BC.

## Conclusion

This rigorous and objective systematic revision suggests that PE is an effective strategy that promotes a positive effect on QoL, cardiorespiratory fitness, and physical functions, including the decrease in BMI and weight of BC survivors. Nonetheless, it should be underpinned that the meta-analyses included show relatively moderate effects, often unsustainable in the long term, and the heterogeneity of each assessment detracts from the power of the evidence, thus requiring these conclusions to be perceived cautiously. Future studies should attempt to disclose the impact of different interventions, frequencies, timings, and adverse events of PE on BC survival outcomes, allowing for refinement of the optimal exercise prescription for each set of patients, aiming to improve the overall QoL.

## Data availability statement

The original contributions and search strategy presented in the study are included in the article/**Supplementary Material**. Further inquiries can be directed to the corresponding author.

## Author contributions

All authors contributed to the manuscript. AJ: conceptualisation, search and trials selection, assessment of the risk of bias, data extraction, and writing of the first draft. IL and PA: search and trials selection, data extraction, and review of the first draft. AC, SV: review of the first draft. AA and LH: conceptualisation and review of the first draft. AM: conceptualisation, search and trials selection, assessment of the risk of bias, data extraction, data synthesis, and writing the first draft. All authors contributed to the article and approved the submitted version.
