# Supplementary material for: Impact of physical exercise programs in breast cancer survivors on health-related quality of life, physical fitness, and body composition: Evidence from systematic reviews and meta-analyses
Source: Front Oncol. 2022 Dec 9;12:955505. doi: 10.3389/fonc.2022.955505 (PMC9782413; doi:10.3389/fonc.2022.955505)
Supplement: Supplementary file 1 [file Table_1.docx]

**Supplementary Materials: Search Strategy**

**PubMed Search Strategy**

Search Dates: December 31, 2021 (Search #1), October 31, 2022 (Search #2)

Final Search: #2

| Search | Query | No. of Results |
| --- | --- | --- |
| #1 | (((((breast cancer) AND (effectiveness OR efficacy OR effective*)) AND (Exercise OR Physical Activity OR Strength Training OR Strength Exercise OR Resistance Training OR Resistance Exercise OR Weight Training OR Weight Exercise OR Aerobic Training OR Aerobic Exercise OR Endurance Training OR Endurance Exercise OR Combined Training OR Combined Exercise)) AND (Meta-Analysis)) AND (Systematic Review)) AND (("2010/01/01"[Date - Publication]: "2021/12/31"[Date - Publication]))  Filters: Full text, Meta-Analysis, Systematic Review, Humans, English | 101 |
| #2 | (((((breast cancer) AND (effectiveness OR efficacy OR effective*)) AND (Exercise OR Physical Activity OR Strength Training OR Strength Exercise OR Resistance Training OR Resistance Exercise OR Weight Training OR Weight Exercise OR Aerobic Training OR Aerobic Exercise OR Endurance Training OR Endurance Exercise OR Combined Training OR Combined Exercise)) AND (Meta-Analysis)) AND (Systematic Review)) AND (("2022/01/01"[Date - Publication]: "2022/10/31"[Date - Publication]))  Filters: Full text, Meta-Analysis, Systematic Review, Humans, English | 29 |
